# Comment on ‘Association Between Dynapenic Obesity and Risk of Cardiovascular Disease: The Hisayama Study’ by Setoyama et al.

**DOI:** 10.1002/jcsm.13658

**Published:** 2024-11-06

**Authors:** Han Wang, Yizhuan Huang

**Affiliations:** ^1^ Acupuncture and Massage Department Affiliated Sport Hospital of Chengdu Sport University Chengdu China

To the Editor:

We write this letter in response to the article [1], ‘Association between dynapenic obesity and risk of cardiovascular disease: The Hisayama study’. The study offers valuable insights into the role of dynapenic obesity as a significant risk factor for cardiovascular disease (CVD) among Japanese community residents, particularly highlighting the importance of weight management and maintaining muscle function in middle‐aged populations. We commend the authors for their contribution and would like to offer some suggestions for further consideration.

First, the study was conducted within a Japanese community where participants share a relatively homogenous genetic and cultural background. As a result, the findings may not be directly applicable to populations with diverse genetic profiles, dietary habits and lifestyle factors in other countries or regions [2]. Future research could benefit from similar studies conducted in various geographical and cultural contexts, exploring how the relationship between dynapenic obesity and CVD risk may vary across different populations. This would enhance the generalizability of the findings and deepen our understanding of these associations across diverse genetic and environmental backgrounds.

Second, while the study included subgroup analyses by age and sex, additional stratifications could provide more precise insights. Future research should consider stratifying participants by levels of physical activity, insulin resistance or diabetes status, types of obesity, medication use, lifestyle factors and socio‐economic status [3, 4, 5]. These analyses would allow for the development of more targeted and personalized intervention strategies aimed at reducing the risk of CVD associated with dynapenic obesity.

Third, although the study's 24‐year follow‐up is commendable, lifestyle factors such as diet, exercise, smoking and alcohol consumption were not tracked dynamically during the follow‐up period. These variables may significantly impact muscle function, obesity and the risk of CVD. We recommend that future studies incorporate multiple data collection points throughout the follow‐up period to monitor changes in lifestyle behaviours, providing a more comprehensive understanding of their role in the development of dynapenic obesity and CVD.

This study underscores the strong association between dynapenic obesity and CVD, emphasizing the critical need for managing muscle strength and body weight in preventing cardiovascular events. As healthcare professionals, we advocate for early screening and identification of dynapenic obesity. Multidisciplinary teams, including internists, nutritionists and physical therapists, can work together to develop personalized, integrated treatment plans. Additionally, enhancing patient education on the risks associated with dynapenic obesity and its impact on CVD prevention will empower individuals to take proactive steps in managing their health.

In conclusion, we greatly appreciate this study's contribution to the growing body of evidence linking dynapenic obesity and CVD. Building upon these findings, we can develop more tailored management strategies that focus on improving muscle function, promoting healthy ageing, reducing the burden of age‐related diseases and ultimately improving the quality of life for older populations.

## Conflicts of Interest

The authors declare no conflicts of interest.
